# Size and shape control of a variety of metallic nanostructures using tilted, rotating evaporation and lithographic *lift-off* techniques

**DOI:** 10.1038/s41598-019-44074-w

**Published:** 2019-05-22

**Authors:** Damien Eschimese, François Vaurette, David Troadec, Gaëtan Leveque, Thierry Melin, Steve Arscott

**Affiliations:** 1 Institut d’Electronique, de Microélectronique et de Nanotechnologie (IEMN), CNRS, The University of Lille, Cité Scientifique, 59652 Villeneuve d’Ascq, France; 2 Horiba France SAS, 231 Rue de Lille, 59650 Villeneuve-d’Ascq, France

**Keywords:** Nanoparticles, Surface patterning

## Abstract

Here, we demonstrate a simple top-down method for nanotechnology whereby electron beam (ebeam) lithography can be combined with tilted, rotated thermal evaporation to control the topography and size of an assortment of metallic objects at the nanometre scale. In order to do this, the evaporation tilt angle is varied between 1 and 24°. The technique allows the 3-dimensional tailoring of a range of metallic object shapes from sharp, flat bottomed spikes to hollow cylinders and rings—all of which have rotational symmetry and whose critical dimensions are much smaller than the lithographic feature size. The lithographic feature size is varied from 400 nm down to 40 nm. The nanostructures are characterized using electron microscopy techniques—the specific shape can be predicted using topographic modelling of the deposition. Although individual nanostructures are studied here, the idea can easily be extended to fabricate arrays for e.g. photonics and metamaterials. Being a generic technique—depending on easily controlled lithographic and evaporation parameters—it can be readily incorporated into any standard planar process and could be adapted to suit other thin-film materials deposited using physical means.

## Introduction

Being able to both precisely and accurately control the shape and dimensions of manufactured objects has always been—and indeed remains—a fundamental challenge in technology at all length scales. Towards the smaller end of this range^1^, metallic sub-micrometre-sized ‘nanostructures’ have numerous applications in many areas of physical sciences, chemical sciences, and life sciences^2–17^. As the fundamental physical behaviour of nanostructures depends on their specific size, structure, composition, shape, and chemistry; the requirements of a particular application can be met by precisely controlling *inter alia* their topography and size—either as unique localized objects or arrays of objects. In order to fabricate such tiny features in a controlled manner, several methods are available—having either a chemical^18,19^ or a physical origin^20^. In the latter context, combining physical vapour deposition^21^ (PVD) and some form of masking to enable openings on the nano-scale^22–24^ facilitates nanostructure formation. The masking can be either lithographic, e.g. electron beam (ebeam) lithography, or bottom-up, e.g. nanoparticle stencils—both approaches lead to ‘shadowing’ effects which determine the size and shape of the resulting object. Historically, lithographically-derived lift-off profiles causing ‘shadowing’ effects have been used for microelectronic circuit patterning^25–28^ and the creation of sharp points^29,30^. Shadowing effects can also be caused by oblique or glancing angle deposition^31–33^ where the wafer is tilted with respect to the evaporation source—these approaches have led to several applications^34,35^. Several types of techniques using e.g. lithographic masking^36–44^, stenciling^45,46^, and bottom-up masking^47–53^ have been used to produce a wide range of nanostructuring. It is in this context that we investigate here the combination of electron beam (ebeam) lithography-derived masking and tilted, rotated thermal evaporation with *lift-off* processing for the novel fabrication of an assortment of metallic nanostructures having rotational symmetry with specific topographies and sizes depending on the particular lithographic mask dimensions *and* the rotation tilt angle employed during the evaporation. We will first describe the fabrication process (masking and evaporation) used here, and then present the resulting nanostructures which are characterized using scanning electron microscopy (SEM) and scanning transmission electron microscopy (STEM). The size and shape of the experimental nanostructures will be compared to the results of a topographic model of the tilted rotated evaporation.

## Results and Discussions

### Fabrication of the samples

In this general context, the following text will describe how to manufacture a range of tailored nanostructures using lithography and tilted, rotated evaporation. Electron beam (ebeam) lithography^54^ is used to fabricate the *lift-off* masks composed of arrays of nanometre-scale openings on commercial silicon wafers (see Methods for processing details). Briefly, in order to achieve this, a flat wafer (3-inch diameter, polished, (100) orientated crystalline silicon) is spin coated with positive-tone ebeam sensitive polymer termed the ‘*resist’*. A copolymer methyl methacrylate/poly(methyl methacrylate) bilayer—commonly referred to as a ‘COPO/PMMA’ bilayer—is used for this purpose (see Methods and Supplementary Information). A focused electron beam—using a commercial ebeam writer (see Supplementary Information)—is then used to locally alter the dissolution rate of the resists during a later resist wet chemical development step which opens quasi-cylindrical shaped ‘nano-holes’ in the resist—revealing the underlying silicon wafer. It is important to remember that the different dissolution rates of the COPO and the PMMA lead to the formation of both ‘overhang’ and ‘undercut’ features (see Supplementary Information)—aspects which are vital for the subsequent *lift-off* of the metallization. The openings or nano-holes have a height *h*, determined by the spin-coated total thickness of the two ebeam resists, and a width *w* governed by the ebeam exposure dose and intended design pattern size. Actually in practice the value of *w* varies from the top of the resist to the bottim—see Supplementary Information—meaning that the real masking is a conical cylinder. The ebeam writing allows several arrays of nano-holes, having several values of *w*, to be formed on a single silicon wafer. This has several advantages for the study: (i) the resist processing conditions are the same for several masking dimensions, e.g. it is known that resist *lift-off* profile is sensitive to development conditions (see Supplementary Information) and (ii) the metallization thickness and tilt angle is the same for several values of *w* allowing a direct nanostructure-to-nanostructure comparison. In the current study, *w* is varied from 400 nm down to 40 nm and *h* is fixed at either 950 nm (COPO/PMMA—900/50 nm) or 500 nm (COPO/PMMA—450/50 nm)—see the Methods and the Supplementary Information for processing details.

### Tilted, rotated evaporation of the samples

**Figure 1:**
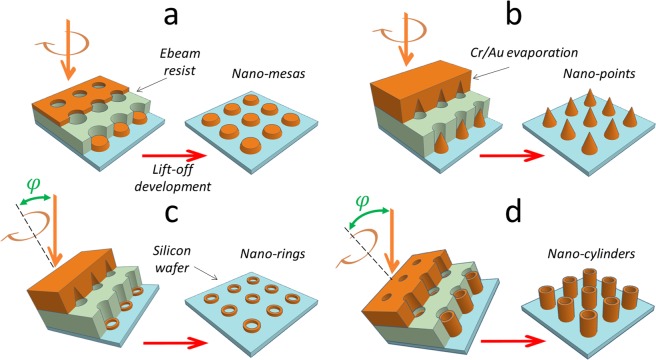
Tilted rotated evaporation via a *lift-off* process involving a *lift-off* shadow mask to produce a variety of nanostructures having rotational symmetry about an axis perpendicular to the wafer surface. The formation of: (**a**) nano-mesas and (**b**) nano-cones at a deposition tilt angle *φ* = 0°. Higher tilt angles *φ* = 0°, lead to the formation of nano-rings (**c**), and eventually nano-cylinders (**d**). The silicon wafer (light blue) is patterned with an electron beam (ebeam) sensitive resist mask (light green) and evaporated with chromium/gold (gold). The resist *lift-off* development step is indicated by the red arrow. The tilt angle *φ* is indicated in (**c**) and (**d**) in green relative to the rotation axis (black dashed line) which is always perpendicular to the wafer surface.

Figure 1 shows a schematic diagram of the samples containing the nano-holes in the bilayer resist being evaporated with a chromium/gold thin film under various conditions. For simplicity the overhang feature is not shown and the cylinders are considered to be perfect right-cylinders.

Before discussing Fig. 1, one needs to be aware that there are several factors which play a role in determining the ultimate structuration. These are the height *h*, width *w*, the resist overhang length, the resist undercut profile in the lower resist of the nano-hole masking (see Supplementary Information), the total thickness *t* of the evaporated matter, the evaporation tilt angle *φ*, the presence of rotation parallel to the axis of the tilt, and the evaporative deposition onto the resist surface, which has both a vertical component *and* a horizontal component—the former effectively increases the ‘height’ of the mask whilst the latter leads to an effective reduction of the opening width of the hole as the evaporation proceeds (see Supplementary Information for more details). Indeed, the latter point can lead to a complete closing of the mask during the evaporation step of the process^55^. It is important to understand that the specific nanostructuration resulting from the deposition depends on the shadowing caused by the subtle interplay of all these parameters—this has been discussed by one of the authors^56^. First, when the evaporation tilt angle is zero (*φ* = 0°) and the opening is relatively large (*w* ≫ *h*), thin film deposition leads to well-known mesa structures—Fig. 1a. However, if the opening is reduced (*w* < *h*)—whilst maintaining a zero tilt angle—then nanometre-scale conic-like structures will be formed due to closing of the resist opening—see Fig. 1b. If we maintain the case where *w* < *h*, then at small tilt angles (0° < *φ* < 5°) evaporation should result in nanometre-scale point-like structures where the specific lateral growth on the mask and subsequent shadowing combine to govern the slope of the profile. Again, maintaining the case where *w* < *h*, at higher evaporation tilt angles (5° < *φ* < 90°), shadowing effects will dominate the deposition and are predicted to lead to the formation of nanometre-scale rings and eventually cylinders. It is important to make a distinction between the fact that the nano-rings are deposited onto the surface of the wafer—resulting from shadowing—see Fig. 1c; whereas the nano-cylinders are deposited onto the sidewalls of the resist—see Fig. 1d. We will now see that such predicted features^56^—and indeed more—can be realized practically by simply controlling bilayer resist coating conditions and evaporation tilt angle.

### Scanning electron microscopy of the samples

**Figure 2:**
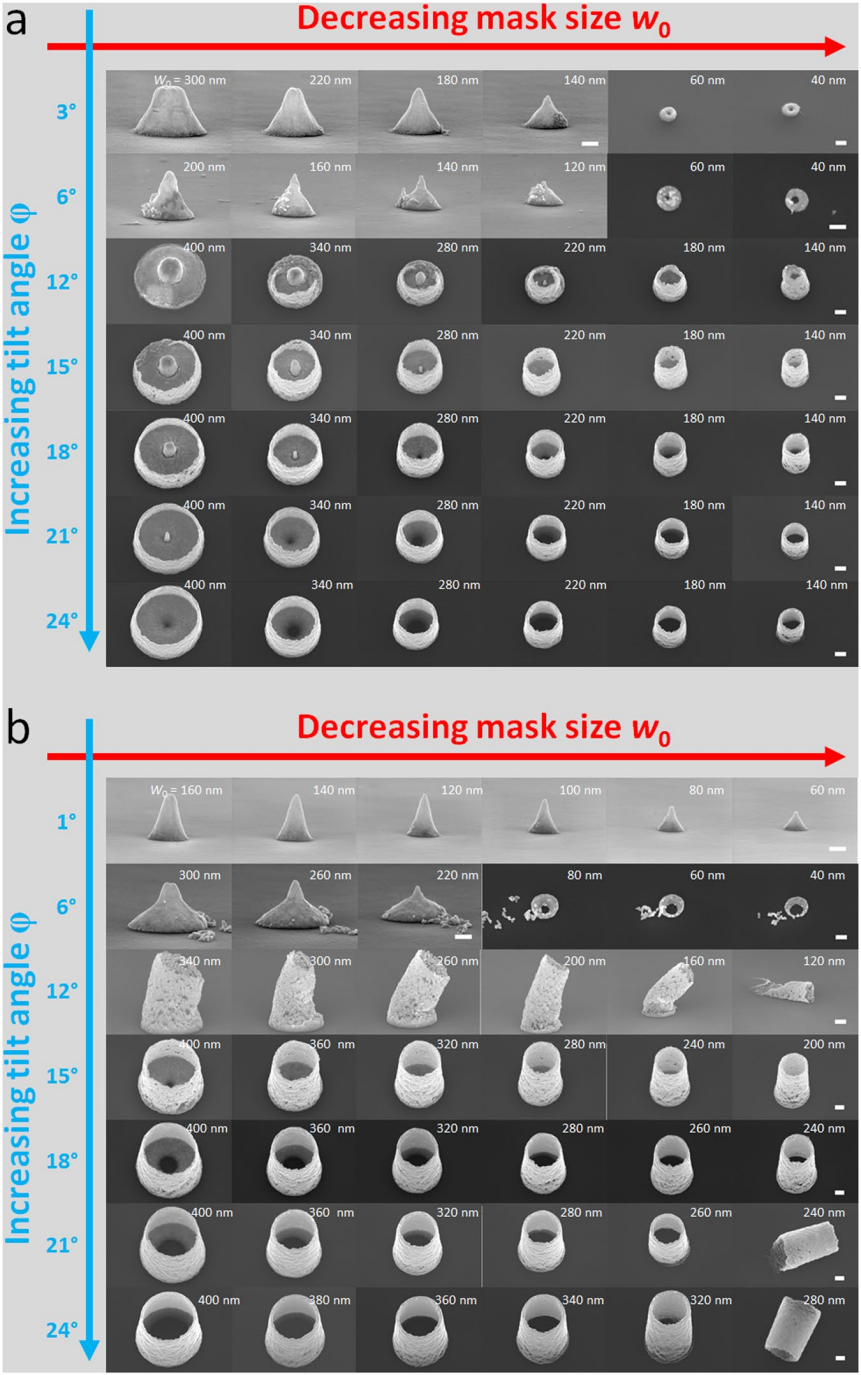
Scanning electron microscopy images showing the variety of metallic nanostructures obtained via the tilted rotating evaporation of gold via a *lift-off* process and shadow mask. In this case an electron beam (ebeam) sensitive ‘resist’ is used. The deposition tilt angle *φ* is varied from 1° to 24° (abscissa axes—blue) and the shadow mask opening width (*w*) from 400 nm to 40 nm (ordinate axes—red). The total thickness of the bilayer ebeam resist (*h*) in this case is 500 nm (**a**) and 950 nm (**b**). In all cases, the evaporated chromium/gold thickness is 300 nm. In all cases the scale bars (white) at the right of the images indicate a length of 100 nm.

Figure 2 shows scanning electron microscopy (SEM) images of experimental gold nanostructures obtained by using a range of evaporation tilt angles *φ* (from 1° to 24°) and resist openings *w* (from 400 nm down to 40 nm)—for a total bilayer resist height *h* of 500 nm (Fig. 2a) and 950 nm (Fig. 2b).

There are a number of specific features and trends which can be observed and remarked upon in the experimental results—which, in addition, agree well with the qualitative predictions given in Fig. 1. Let us consider first the experimental results obtained using a resist mask height *h* = 500 nm—see Fig. 2a. First, at low tilt angles (*φ* = 3° and 6°), and relatively large mask openings (300 nm to 120 nm), one finds the expected well-known mesa structures having a sloped edge due to the lateral deposition onto the mask edge which causes progressive shadowing. The flat-topped mesa features becoming sharp pointed structures below a certain critical mask opening width. When the mask opening becomes small, the predicted nano-ring features appear. For example, at a tilt angle of 3° the inner and outer diameters of the nano-rings increasing if the tilt angle is increased to 6°. When the tilt angle is increased to 12° a flat-topped feature appears which has a thinner base than a ‘mesa’. Interestingly, as the mask opening width is decreased (at *φ* = 12°) the central flat-top region becomes a small ‘spike’ and is surrounded by an emerging nano-cylinder. The appearance of the nano-cylinders is caused by a usually unwanted phenomenon, commonly called ‘ears’, which is observed in photolithographic *lift-off* techniques—it is generally associated with the deposition of matter on the sidewalls of a resist profile^57^, and remedied using a ‘scrub’ with cleanroom paper and a solvent. As the mask opening width is decreased, the width of the nano-cylinder diminishes. We can now see how increasing the tilt angle affects a specific feature size. As *φ* is increased from 15° to 24 °, at a large mask opening (*w* = 400 nm) the central flat-top region becomes a nano-spike (at *φ* = 21°) and becomes a ‘hole’ to form a nano-ring on the wafer surface (at *φ* = 24°). In all cases—at *w* = 400 nm—there is a nano-cylinder surrounding, and indeed attached to, the surface feature. This trend is seen as the feature size is reduced, with the transition from nano-spike to ‘hole’ happening at small tilt angle. Interestingly, all feature sizes studied (*w* = 400 nm to 40 nm) result in nano-cylinders having diminishing diameter. Indeed, a close look at the SEM images reveals that the thickness of the nano-cylinders increases as the tilt angle is increased—as would be expected qualitatively.

Let us now look at Fig. 2b in this case the total resist thickness *h* is 950 nm. It is apparent that similar trends are observed compared to the smaller value of *h*. First, classic nano-conic-like feature formation occurs at low tilt angle (3°) and for all feature sizes. These types of cones are called Spindt-like^29^ features—similar to those fabricated by Kontio *et al*.^39^ and Schaffer *et al*.^41^. Next, we observe nano-spike formation at low angle (*φ* = 6°) intermediate feature size (*w* = 320 nm). At *φ* = 6°, nano-ring formation occurs at a lower feature size (*w* < 100 nm). For *φ* = 6°, we see the vestiges of nano-cylinder formation – but they are clearly not robust to the *lift-off* process—which involves a heated solvent bath. Again, we can focus on specific features sizes as the tilt angle *φ* is increased. At larger feature sizes, we observe nano-ring formation having an inner diameter which increases with tilt angle—the ring is connected to an accompanying nano-cylinder whose wall thickness increases with tilt angle. At intermediate feature size the nano-ring disappears at lower tilt angle. At low feature sizes, the nano-cylinder is not attached to the wafer surface. The nano-cylinders become detached or *released* from the surface (during the *lift-off* solvent bath) at larger feature size as the tilt angle is increased. A number of ‘movies’ can be found in the Supplementary Information which illustrate well how the topography of the nanostructures changes with evaporation tilt angle and lithographic feature size and resist height. It can be observed that the nano-cylinders fabricated at a tilt angle or *φ* = 12° [Fig. 2b] are porous and not rigid enough to survive the *lift-off* process. It can also be observed that the porosity along the nanocylinder seems to change along its length—this is apparent in Fig. 2b at tilt angles of 21° and 24° for the smallest feature sizes.

### Scanning transmission electron microscopy (STEM) of the nanostructures

**Figure 3:**
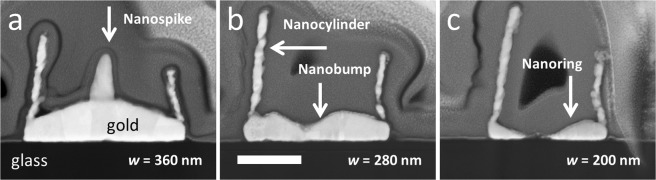
Scanning transmission electron microscopy (STEM) images of 3 nanostructures fabricated using tilted, rotated evaporation. The resist opening widths are: (**a**) 360 nm, (**b**) 280 nm, and (**c**), 200 nm. The cross-section of the nanostructures was made using Focused ion beam (FIB) etching (see Supplementary Information for details). The evaporation tilt angle *φ* = 15°. The bilayer resist thickness *h* = 500 nm. The white scale bar in (**b**) indicates 200 nm.

Scanning electron microscopy enables an overall view of the individual nanostructures (see Fig. 2) and can provide information concerning certain dimensions of the nanostructures e.g. cylinder heights and diameters. However, SEM cannot be used to measure topographic details of the structures *inside* the nano-cylinders e.g. the profile information of the nano-rings and nano-spikes with modifying the structures. In order to obtain this data focused ion beam (FIB) techniques can be used to produce cross-sectional nanostructured samples for scanning transmission electron microscopy (STEM)—see Supplementary Information. Figure 3 shows the results of the STEM measurements.

The STEM allows one to measure with some accuracy the topography of the deposition on the wafer surface inside the nanocylinder which can be compared to topographic modelling—see Supplementary Information. One can also obtain an approximate measurement of the thickness of the nanocylinders (~15–25 nm). The granularity and the non-uniformity of the deposition on the sidewall are apparent in Fig. 3. As will be seen in the following section, a topographic model is able to successfully predict these features and their dimension—see Supplementary Information.

Some important practical issues need to be discussed concerning the experimental setup. First, concerning the uncertainty and precision of the nominal tilt angle *φ*, let us consider the practical set up that we used for the experiments (see methods and Supplementary Information). The crucible evaporation source to wafer distance is ~50 cm, the crucible diameter is ~1.5 cm, and the ebeam raster ‘hotspot’ diameter is typically ~0.5 cm (for gold). The angle variation from one side of the hotspot to the other is therefore given by tan^−1^0.5/50 ≅ 0.6°. In other words, the uncertainty in the nominal angle stated in the manuscript—due to the rastering effect of the ebeam on the crucible—is of the same order as the angle uncertainty (precision) of the wafer angle which sets the inclination—given by the manufacturers (0.5°). This aspect of ‘blurring’ of features using a stencil or *lift-off* mask has been considered and discussed by Vazquez-Mena *et al*.^58^. They derive the following equation (Eq. 1) to calculate the blurring of matter produced using a stencil or lift-off mask which is not in contact with the wafer:

1 $${l}_{b}\approx \frac{{h}_{r}{w}_{s}}{{d}_{sw}}$$

where *l*_b_ is the characteristic blurring length of the deposited matter, *h*_*r*_ is the height of the photoresist, *w*_*s*_ is the width of the evaporation source, and *d*_*sw*_ is the distance from the evaporation source to the wafer surface. If we take the practical values quoted above and a resist height of 950 nm and 500 nm, one estimates the expected characteristic blurring length *l*_b_ to be 9.5 nm and 5 nm respectively.

Another important point concerning the study here is achieving rotational symmetry of the resulting nanostructures. Accomplishing rotational symmetry depends on the mask closing due to the deposition of matter and the number of rotations required to achieve the mask closing. First, if mask does not close during the evaporation—then rotational symmetry is achieved if the number of revolutions *n* of the wafer during the evaporation is an integer. Second, if the mask does close during the evaporation—then rotational symmetry is achieved if the mask-closing *exactly coincides* with the completion of a whole wafer revolution. However, and thirdly, a *high degree* of rotational symmetry can be achieved if the following inequality in Eq. 2 is met during the evaporation:

2 $$v\gg \frac{1}{{t}_{c}}$$

where *v* is the rotational speed of the wafer (revolutions s^−1^) and *t*_*c*_ is the characteristic fabrication time (s) of the nanostructure, i.e. the time from the evaporation beginning to the mask closing. This characteristic time is given by Eq. 3:

3 $${t}_{c}={h}_{s}/\eta $$

where *h*_*s*_ is the height of the nanostructure and *η* is the evaporation rate (m s^−1^) of the matter. We can take some of the practical values from the study to evaluate the inequality in Eq. (2). The rotational speed *v* is 5.2 revolutions per minute (rpm), the evaporation rate *η* equals 0.5 nm s^−1^, and the smallest *h*_*s*_ can be taken to be of the order of 50 nm—the value of *t*_*c*_ in this case is 100 s. In this case, the above inequality is met by a factor of ~8, suggesting approximate rotational symmetry for resulting shapes having *h*_*s*_>50 nm. The authors note that it would be interesting to explore the case where the inequality is not satisfied—in principle tuneable shapes having an absence of rotation symmetry would be expected. Concerning the film thickness uniformity of the deposition across the wafer—if we consider the size of the silicon wafer (diameter ~7.62 cm) and the evaporation source-to-wafer distance (~0.5 m) then the thickness uniformity is near unity^59^.

The experimental results obtained here [Figs 2, 3] using tilted rotating evaporation can be compared to some of those found on the literature. Kontio *et al*.^39^ and Schafer *et al*.^41^ used a Spindt-type shadowing approach (no indication of tilting or rotation during evaporation given) to fabricate arrays of metallic nanocones. Kosiorek *et al*.^47^ coupled shadow nanosphere lithography with rotated, angled evaporation to create a range on nanometre-sized patterns on the wafer surface. Gwinner *et al*.^48^ created nanometre-sized spilt ring resonator patterns on a wafer surface using rotated shadow evaporation via nanospheres. Dickey *et al*.^38^ made use of very high aspect ratio support features and tilted, rotating evaporation to produce dense arrays of nanotubes having rotational symmetry. The use of a nanostencil and rotation during pulsed laser deposition can also produce nano-scale patterning^45,46^. Yu and Chou^37^ used static-angled deposition of nanometre-dimension 3D triangular features.

### Topographical modelling of the nanostructures

**Figure 4:**
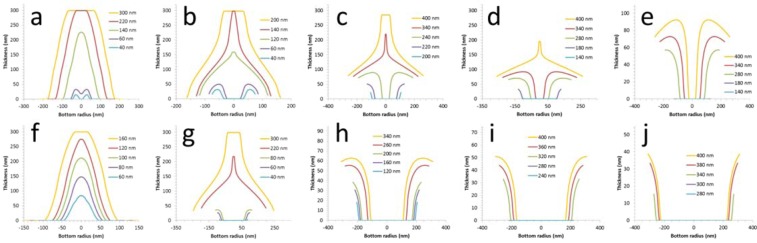
Predictions of the topographic modelling of the nanostructures. (**a**–**e**) *h* = 500 and, (**f**–**j**), *h* = 950. The tilt angle *φ* is < 5° (**a**,***f***), 6° (**b**,**g**), 18° (**c**,**h**), 21° (**d**,**i**), and 24° (**e**,**j**). The resist opening *w* is varied from 400 nm down to 40 nm—see individual legend. Note (**a**) is at 3° and (**f**) is at 1°.

**Figure 5:**
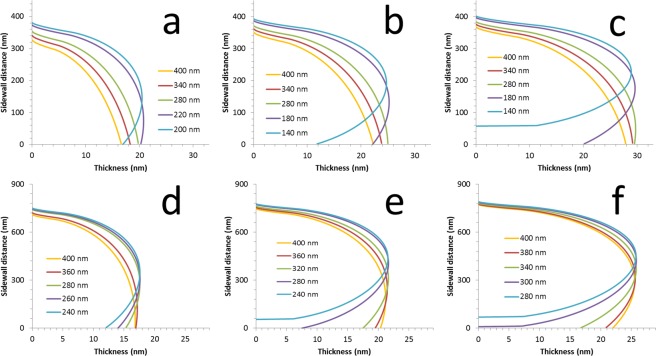
Results of the topographic modelling of the deposition on the resist sidewalls. (**a**–**c**), *h* = 500 and, (**d**–**f**), *h* = 950. The tilt angle *φ* is 18° (**a**,**d**), 21° (**b**,**e**), and 24° (**c**,**f**). The resist opening *w* is varied from 400 nm down to 140 nm—see individual legend.

Despite the intuitive and qualitative description of the fabrication (Fig. 1), a mathematical model which can predict the specific topography of the nanostructuring needs to be used which takes into account the factors described above (e.g. mask dimensions, tilt angle, deposition onto masking…). An analytical model^56^ can be developed to achieve this by making some assumptions concerning the evaporation process and setup (see Supplementary Information). Interestingly, such modelling approaches can be found in other areas of science—at much larger length scales^60^! The resist overhang length and undercut dimensions were extracted from the experimental results and used in the model. In this way, the specific nanostructuration resulting from the shadowing can be predicted. These results of the topographic modelling are shown in Figs 4 and 5.

If we compare these figures to the experimental results in Fig. 2, it is clear that the topographic modelling of the nanostructuration predicts all the trends seen in the experimentation. More so, the modelling is also able to successfully predict the dimensions e.g. nano-ring diameters and heights, and nano-cylinder wall thicknesses. It can be seen that the model predicts all the *trends* seen in the experimentation, and secondly, is able to predict *specific* conditions (i.e. resist dimensions and evaporation angle) where distinct changes are seen in the experimental nanostructuration. By comparing Fig. 2 with Figs 4, 5 let us talk about some specific examples. First, the precise shape of the nano-mesas is predicted at large opening and low tilt angle. Nano-ring formation is predicted as the opening is reduced to 60 nm at low tilt angle. The specific nano-ring dimensions are successfully predicted as the tilt angle *φ* is increased from 3° to 6° for *h* = 500 nm—with nano-ring formation halting at higher tilt angles as is seen in the experimentation.

Nano-spike formation (with and without an associated nano-cylinder) is predicted at the experimentally used resist and tilt conditions. This is best seen by comparing Fig. 4d with Fig. 4e. At *w* = 400 nm a spike is formed when passing from a tilt angle *φ* of 24° to 21°—precisely as observed in the experiments. Nano-cylinder height diminished with reducing tilt angle—as is seen in the experimental results—and increases at a given tilt angle as the resist opening is diminished. The modelling also predicts the non-uniformity of the deposition on the sidewall which is apparent in the experimental results. Indeed, it can be observed in the experimental results that the apparent porosity some nano-cylinders varies over the length of the feature—this seems to suggest a variation of nano-cylinder thickness as is suggested by the modelling of the sidewall deposition.

## Conclusions

In conclusion, it is shown that tilted, rotated evaporation using small *lift-off* profile bilayer resist masking can result in a range of 3D nanostructures whose critical dimensions are less than the resist feature size. The specific nanostructures revealed by the study vary from nano-spikes and nano-bumps to nano-rings and nano-cylinders. The nanostructures have rotational symmetry about an axis perpendicular to the surface of the wafer due to (i) the rotation during evaporation and (ii) the cylindrical nature of the mask opening. Concerning this last point, the authors note that if non-cylindrical masking would result in nanostructures not having rotational symmetry if this was desired. The specific size and topography of the experimentally-obtained nanostructures agrees well with a topographic modelling of the tilted rotating evaporation process. The generic fabrication process of the structures uses top-down methods which are—at least in principle—compatible with most micro and nanofabrication processes for microelectronics and micro/nanoelectromechanical systems. Finally, despite using metallic physical vapour deposition here as a proof-of-concept demonstration, in principle any evaporative material or indeed combinations of materials—e.g. to form evaporated heterojunctions^61^ having a specific shape—could be used to form such features.

## Methods

### Fabrication of the samples

3-inch diameter single-crystalline, (100) orientated silicon wafers (Siltronix, France) p-type boron resistivity (5–10 Ω cm) were used for the fabrication. Prior to resist deposition, the silicon wafers were subjected to an ‘RCA’ clean^62^. Two electron beam (ebeam) resists COPOPOLYMER or ‘COPO’ and PMMA were used to form a bi-layer lift-off profile. The processing parameters were optimised for the study—details can be found in the Supplementary Information. The thermal evaporation was performed in a commercial MEB 550 S electron beam evaporation system (Plassys, France) at <10^−7^ mbar. The machine specifications enable the sample holder to be tilted at an angle – relative to the evaporation crucible – of 0° to 25°. The sample holder is rotated at a speed of 5.2 rpm during the evaporation. Following removal of the samples from the evaporator, the *lift-off* process is completed by immersion of the samples in a commercial resist remover SVC-14 (Seidler Chemical Co, USA) at 70 °C. The samples were observed using an Ultra-55 (Zeiss, Germany) scanning electron microscope. A Strata dual beam 235 focused ion beam (FIB) (FEI, USA) was used to produce samples for scanning transmission electron microscopy (STEM)—see Supplementary Information.

## Supplementary information

Supplementary Infomation

500nm 3deg

500nm 6deg

500nm 12deg

500nm 15deg

500nm 18deg

500nm 21deg

500nm 24deg

950nm 1deg

950nm 6deg

950nm 6deg_1

950nm 15deg

950nm 18deg

950nm 21deg

950nm 24deg
