# Supplementary material for: Size and shape control of a variety of metallic nanostructures using tilted, rotating evaporation and lithographic lift-off techniques
Source: Sci Rep. 2019 May 22;9:7682. doi: 10.1038/s41598-019-44074-w (PMC6531472; doi:10.1038/s41598-019-44074-w)
Supplement: Supplementary file 1 — Supplementary Infomation [file 41598_2019_44074_MOESM1_ESM.pdf]

# Supplementary Information

## 'Size and shape control of a variety of metallic nanostructures using tilted, rotating evaporation and lithographic *lift-off* techniques'

Damien Eschimese*†, François Vaurette*, David Troadec*, Gaëtan Leveque*, Thierry Melin*, and Steve Arscott*

**Institut d'Electronique, de Microélectronique et de Nanotechnologie (IEMN), CNRS, The University of Lille, Cité Scientifique, 59652 Villeneuve d'Ascq, France.*

†*Horiba France SAS, 231 Rue de Lille, 59650 Villeneuve-d'Ascq, France.*

### 1. Detailed description of the nanofabrication process for the nanostructures.

We describe here a detailed description of the experimental lithographic process developed for the fabrication of the nanostructures shown in the main article. The process involves wafer cleaning, resist deposition by spin-coating, the electron beam exposure, the development of the resist, metallic deposition and a '*lift-off*' process. First of all, 3-inch diameter, polished crystalline (100) orientated silicon wafers (Siltronix, France) are cleaned using an RCA procedure.[1] The wafer surfaces are then dehydrated a hotplate at a temperature of 180°C for 10 minutes. Following surface dehydration, a bilayer electron beam (ebeam) sensitive *resist* combination is spin coated onto the wafer surfaces to enable the required overhang and undercut features for a *lift-off* process. The first resist layer is composed of a 450 nm thick layer PMMA EL13% (MicroChem, USA) and is deposited onto the wafer surfaces by spin-coating (RC8, SÜSS MicroTec SE) at 4000rpm/1000rpm $s^{-1}$/12s to obtain 450 nm and at 2250rpm/1000rpm $s^{-1}$/12s to

obtain 900 nm; followed by an homogenisation step (500rpm/1000rpm $s^{-1}$/6s). Post-baking of the PMMA is then performed also in two steps in order to prevent thermal shock (1 min at 80°C followed by 10 min at 180°C). A second resist layer (PMMA 950K diluted 5/3 Anisole, MicroChem, USA) is then deposited using spin coating onto the first PMMA resist to form the 'overhang' feature. The second resist layer has a thickness of 50 nm (4000rpm/1000rpm $s^{-1}$/12s) followed by an homogenisation step (800rpm/1000rpm s-1/8s) and a resist pre-bake of 1 min at 80°C followed by 10 min at 180°C—again to avoid potential thermal shock. The bilayer resist coated wafers are then loaded into the electron beam lithography chamber (Raith EBPG 500 Plus at 100kV). Following a certain pattern guideline created with software (LayoutEditor), an electron beam (1500 µC $cm^{-2}$ fixed dose, 1 nA beam current) is used to locally expose each wafer. The mask design was circles having diameters ranging from 40nm to 400nm. In order to remove the exposed part of the resist and form the overhang and undercut features required for the eventual lift-off step a chemical development of the bilayer is performed. The exposed resist bilayers are developed using a mix of VLSI grade methyl isobutyl ketone (MIBK) and isopropyl alcohol (IPA) with a volume ratio of *v*/*v* = 1/2 for 60 s. The wafers are then rinsed in pure IPA and dried with dry nitrogen. The patterned silicon wafers are then loaded into a MEB 550S electron beam evaporation system (Plassys, France) at < $10^{-7}$ mbar for a chromium/gold deposition using tilted, planetary-rotated, thermal (beam) evaporation. A 5 nm thick 'adhesion layer'[2] of chromium is first deposited at ~0.2 nm $s^{-1}$. The authors note that the choice of adhesion metals is important for potential photonic applications[3]—chromium is also relatively chemically robust.[4] Following this, a 300 nm thick gold layer is deposited at an evaporation rate of ~0.5 nm $s^{-1}$. During evaporation of both elements, the wafers can be tilted with a relatively good accuracy (±0.5°) and rotated (5.2 rpm). The evaporated wafers are removed from the evaporator and a lift-off procedure is conducted. This consists of removing the bilayer resist using a Remove PG (Microchem, USA) bath at 70°C for 2h. In order to complete the process, the samples are rinsed in acetone and IPA and dried using dry nitrogen.

## 2. Main nanostructures revealed by the study

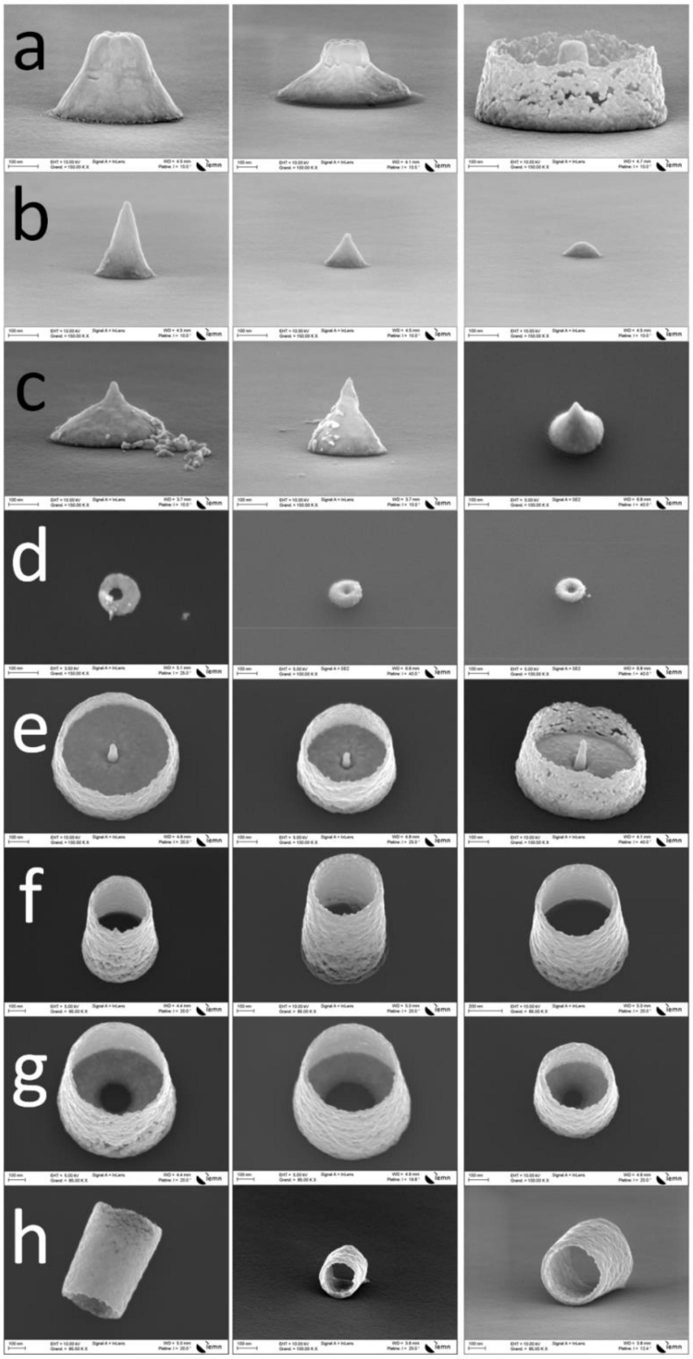

**Supplementary Figure 1 The different types of nanostructure topography revealed experimentally by the study. a,** nano-mesa structures—which can be surrounded by a nano-cylinder. **b**, Nano-cones which diminish to nano-bumps. **c,** Nano-spikes having a flat base. **d,** nano-rings. **e,** Nano-spike combined with a nano-cylinder. **f,** Nano-cylinders without deposition on the wafer surface, **g,** Nano-cylinders combined with a nano-ring. **h,** Detached or *released* nano-cylinders which can be recuperated in solution after *lift-off*.

Supplementary Figure 1 shows the different nanostructures revealed by the study. Depending on the evaporation tilt angle, resist opening $w$ and height $h$, and resist overhang and undercut profile, the lift-off technique can result in 8 specific types of features. These are nano-mesas (which can be associated with a nano-cylinder, nano-cones having various aspect ratios, nano-spikes composed of a shape feature on a flatter base, nano-rings—having a 'sliced bagel' profile—on the wafer surface, nano-spikes having an associated nano-ring, nano-cylinders having no surface deposit, nano-cylinders having nano-rings at the interior, and *released* nano-cylinders.

## 3. Experimental nano-ring dimensions

Supplementary Figures 2 and 3 shows plots of the experimentally obtained nano-ring dimensions. The nano-ring heights are shown in Supplementary Figure 2 and the aspect ratio of the nano-rings (height/average width) are plotted in Supplementary Figure 3.

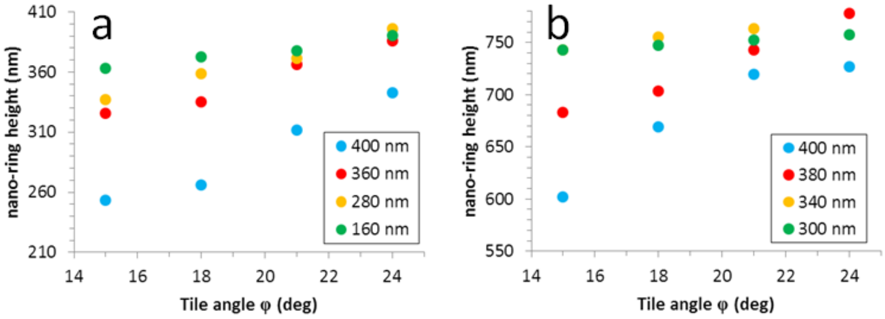


**Supplementary Figure 2 Experimental nano-ring height as a function of evaporation tilt angle and resist opening diameter $w$. a,** $h$ = 500 nm. **b**, $h$ = 950 nm.

Supplementary Figure 3 shows Experimental nano-ring aspect ratio as a function of evaporation tilt angle and resist opening aspect ratio ($h/w$). The nano-ring aspect ratio is defined as the nano-ring height divided by the average nano-ring width.

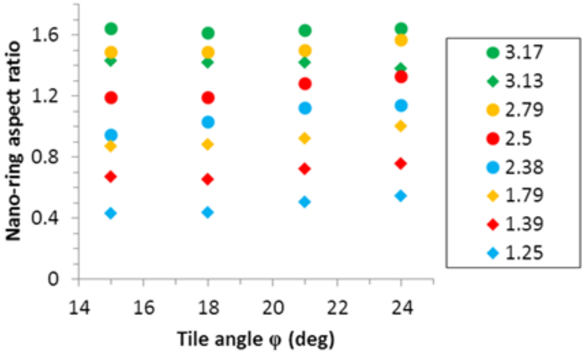


**Supplementary Figure 3 Experimental nano-ring aspect ratio as a function of evaporation tilt angle and resist opening aspect ratio ($h/w$).** The filled circular data is for $h$ = 500 nm whilst the filled diamond data is for $h$ = 950 nm.

## 4. Experimental determination of the resist *lift-off* profile overhang length Δ and undercut profile slope $\beta$.

The experimental results—which demonstrate nano-ring formation at higher tilt angles—enable an extraction of the overhang length Δ and the resist undercut angle $\beta$ which can be used in the topographic modelling (see below). The values of Δ and $\beta$ are calculated using simple geometry assuming a non-right cylinder. Supplementary Figure 4 shows the extracted undercut length Δ and the resist undercut angle $\beta$ of the bilayer resist as a function of resist opening *w* for the two different bilayer resist thicknesses.

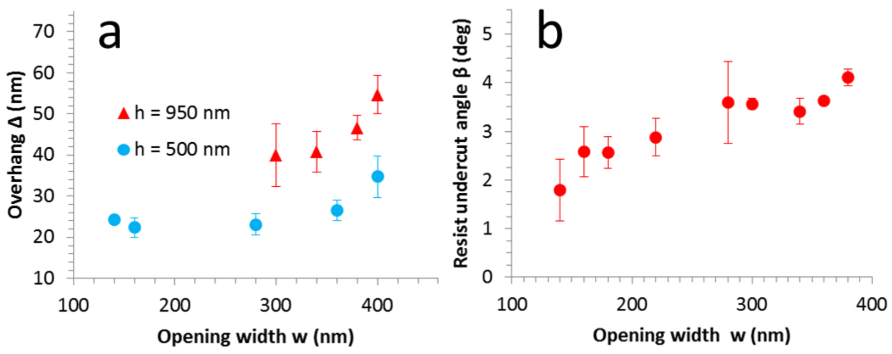


**Supplementary Figure 4 Experimental features of the lift-off profile. a**, The overhang length Δ, and **b**, resist undercut angle $\beta$ as a function of resist opening dimension $w$ and resist bilayer thickness $h$.

There are some interesting points to note here. First, the overhang length Δ is lower for a lower resist thickness as the lower resist is exposed to the solvent for less time. This length, which is highly sensitive to processing conditions, has a key role to play concerning the nanocylinder height. Second; the overhang length Δ increases with the opening width $w$. This can be explained by realizing that for a larger opening, more of the underlying resist material needs to be removed. Third, the underlying resist undercut profile angle increases with opening width $w$. This ca be explain by the difference in ebeam

dose for a positive resist—the larger features require a larger dose which will result in amplified edge effects.

## 5. Scanning transmission electron microscopy (STEM) of the samples.

Some of the individual nanostructures containing deposition on the bottom of the wafer were studied using scanning transmission electron microscopy (STEM). The STEM was performed on 'TEM-prepared' samples fabricated using focused ion beam (FIB) techniques. FIB preparation of the TEM-prepared samples and the subsequent STEM analysis was performed in a Strata dual beam 235 (FEI, USA). First, a thin platinum layer is deposited via ebeam-assisted techniques locally on each nanostructure in order to protect the gold nanostructures from the ion beam etching. Second, a narrow dashed platinum line is deposited (ebeam assisted deposition) along the centre of seven nanostructures. Two carbon (ion beam assisted deposition) cross patterns, spaced by 15 µm, are then deposited on the dashed Pt line. Following this, a platinum deposition (ebeam assisted) is performed to protect all depositions from the ion beam. A combination of carbon and platinum enables scanning electron microscopy (SEM) observation contrast during fabrication of the TEM-preparation samples. The two carbon crosses allow one to localise the dashed platinum line running at the centre of the nanostructures during FIB etching. Then, intense (a 3 nA beam current) FIB etching parallel to—and in proximity to—the whole of the line is performed to a thickness of ~1.5 µm. Following this, FIB etching at a lower beam current (100 pA) FIB etching is conducted on each nanostructure individually to a thickness of <100 nm. Observation of the nanostructures' TEM-preparation samples is performed using SEM at 30 kV in STEM (Transmission) mode.

## 6. Modelling of the topographic deposition of the nanostructure formed by the tilted evaporation.

The experimentally obtained nanostructuration presented in the article, i.e. nano-mesas, nano-spikes, nano-rings, nano-cylinders, nano-bumps, can all be predicted using a simple analytical modelling approach which makes some approximations in order to model thermal evaporative deposition via an 'idealized' *lift-off* resist profile—the specific details of the derivation of the model have been published by one of the authors.[5] This approach can be employed due to a certain rotational symmetry of the masking. This is in contrast with numerical approaches[6,7] which can deal with—at least in principle—more complex geometries but may be more cumbersome to implement. The idealized *lift-off* profile, used for the topographic modelling of matter deposited on that wafer surface and sidewalls, is shown below in Supplementary Figure 5.

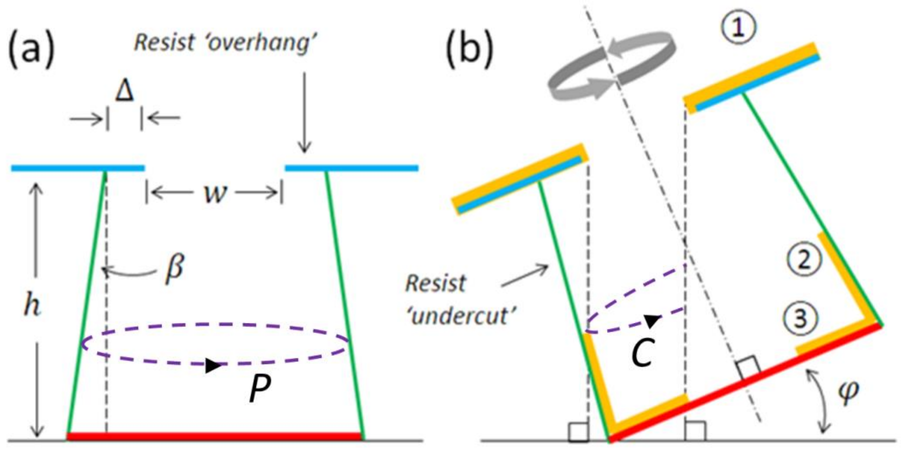


**Supplementary Figure 5 Schematic diagram of an idealized resist-based *lift-off* mask involving overhang and undercut features. a,** Relevant dimensions used in the mathematical modelling. **b,** The *lift-off* mask when tilted and being deposited with matter. There is deposition on the mask ① and the formation of nano-cylinders ② and nano-rings ③ on the 'undercut' sidewalls (green) and the wafer

surface (red). The dashed purple ellipse—in (a)—and dashed purple arc—in (b)—indicate the perimeter $P$ at a given hieight and the chord $C$ of that perimeter that is *not* in the shadow of the mask. The ratio $C/P$ is proportional to the amount of matter deposited on that perimeter per rotation of the evaporation.

We can consider the resist opening to be composed of an overhang (blue) and a sloped undercut (green) feature having dimensions shown in Supp. Fig. 5. A linear slope approximation is used here. The experimentally obtained values of the overhang dimension $\Delta$ and the undercut angle $\beta$ can be used in the modelling. The simple model considers the parts of the wafer surface (red) and resist sidewalls (green) that are not in the shadow of the mask (blue). By assuming rotational symmetry, the lengths of projected chords $C$ onto these parts can be evaluated analytically. The total perimeter $P$—either on the surface or on the sidewall can also be written down analytically. The amount of matter that is deposited onto that perimeter is thus simply proportional to the ratio $C/P$.

In order to account for the observed nanotubes having a sloped edge—due to the practical sloped undercut of the bottom resist—the model[5] has been adapted to account for this. This is a relatively straightforward task in that the slope angle $\beta$ shown in Supplementary Figure 5 simply leads to a linear increase in the perimeter $P$ of the opening towards the bottom of the opening. It is relatively trivial to account for this mathematically. Concerning the assumptions of the model—first, we assume that the evaporation tilt angle is constant during the deposition of the matter and that the mask rotates about the axis of the opening. We assume that the evaporation source-to-wafer distance is much larger than the mask dimensions and the spacing between the features on the wafer. We also assume that the matter is deposited evenly over the patterns[8] and that the evaporated matter arrives in straight lines from the source. We assume that the evaporation rate and deposition rate of the matter are constant

during the evaporation. Finally, issues such as the granularity of the evaporated matter, the migration of species on the wafer surface, or 'blurring'[9] via a stencil mask are not taken into account.

Supplementary Figure 6 shows results of the modelling of the nanostructuring corresponding to the conditions shown in Figure 3 of the article. The model predicts the experimental nanostructuring and the specific topographic dimensions.

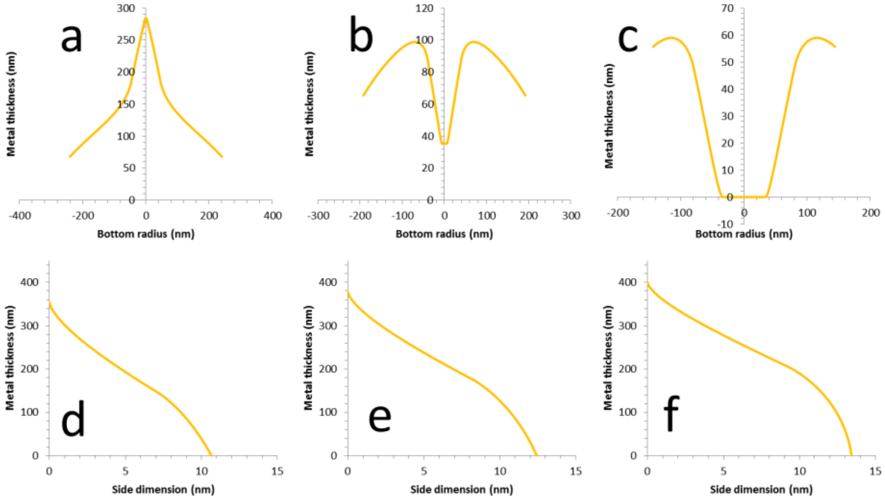


**Supplementary Figure 6 Topographic modelling of the experimental nanostructuring shown in Fig. 3 of the article.**

## Supplementary references

1. Kern, W. The Evolution of Silicon Wafer Cleaning Technology. *J. Electrochem. Soc.* **137**, 1887–1892 (1990).
2. Heavens, O. S. Some factors influencing the adhesion of films produced by vacuum evaporation. *J. Phys. Radium* **11**, 355–360 (1950).

3. Siegfried, T., Ekinci, Y., Martin, O. J. F. & Sigg, H. Engineering Metal Adhesion Layers That Do Not Deteriorate Plasmon Resonances. *ACS Nano* **7**, 2751–2757 (2013).
4. Williams, K. R., Gupta, K. & Wasilik, M. Etch rates for micromachining processing-Part II. *J. Microelectromechanical Syst.* **12**, 761–778 (2003).
5. Arscott, S. On evaporation via an inclined rotating circular *lift-off* shadow or stencil mask. *J. Vac. Sci. Technol. B* **37**, 011602–12 (2019).
6. Oldham, W. G., Neureuther, A. R., Chiakang Sung, Reynolds, J. L. & Nandgaonkar, S. N. A general simulator for VLSI lithography and etching processes: Part II—Application to deposition and etching. *IEEE Trans. Electron Devices* **27**, 1455–1459 (1980).
7. Baumann, F. H. *et al.* Multiscale Modeling of Thin-Film Deposition: Applications to Si Device Processing. *MRS Bull.* **26**, 182–189 (2001).
8. Ohring, M. *Materials Science of Thin Films.* (Elsevier, 2001).
9. Vazquez-Mena, O. *et al.* Analysis of the blurring in stencil lithography. *Nanotechnology* **20**, 415303–10 (2009).
