# Supplementary figures and images for: Size and shape control of a variety of metallic nanostructures using tilted, rotating evaporation and lithographic lift-off techniques
Source: Sci Rep. 2019 May 22;9:7682. doi: 10.1038/s41598-019-44074-w (PMC6531472; doi:10.1038/s41598-019-44074-w)

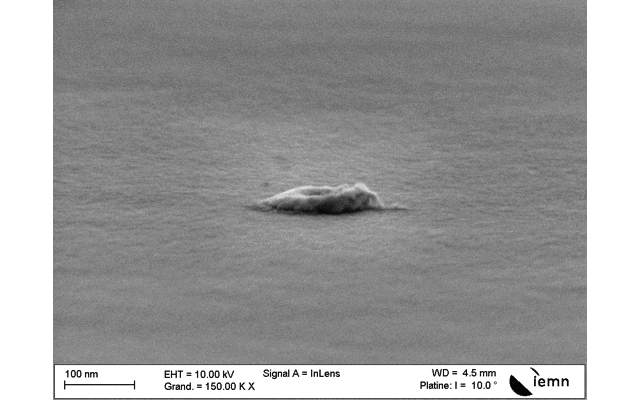

Supplement: Supplementary file 2 — 500nm 3deg [file 41598_2019_44074_MOESM2_ESM.gif]

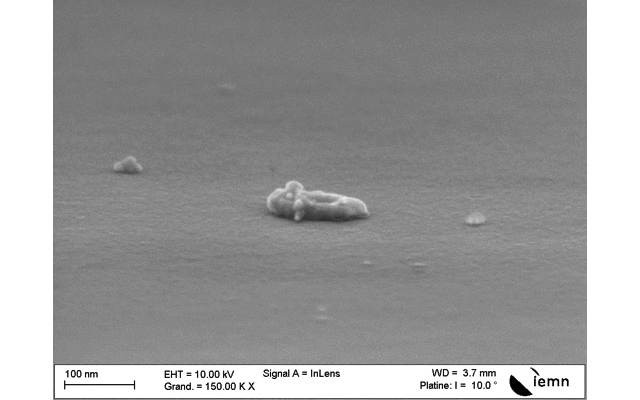

Supplement: Supplementary file 3 — 500nm 6deg [file 41598_2019_44074_MOESM3_ESM.gif]

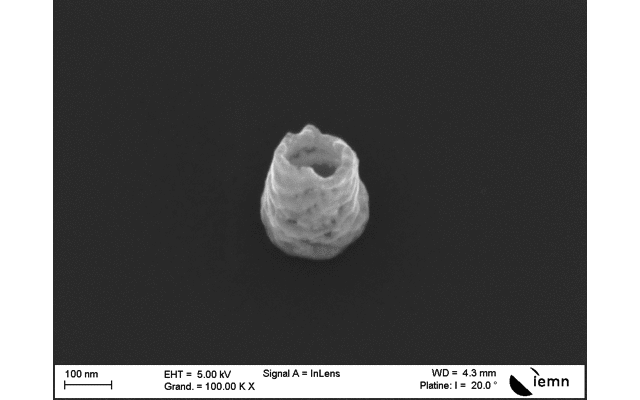

Supplement: Supplementary file 4 — 500nm 12deg [file 41598_2019_44074_MOESM4_ESM.gif]

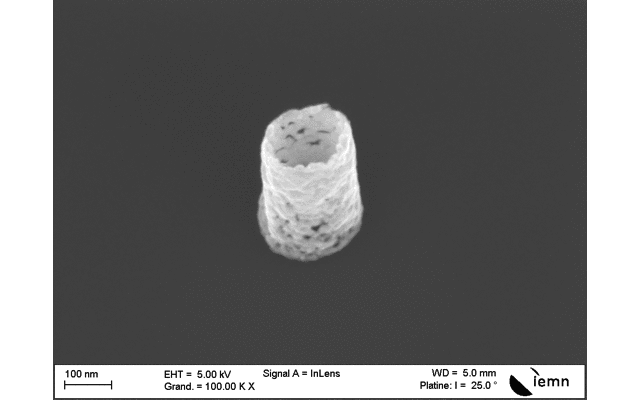

Supplement: Supplementary file 5 — 500nm 15deg [file 41598_2019_44074_MOESM5_ESM.gif]

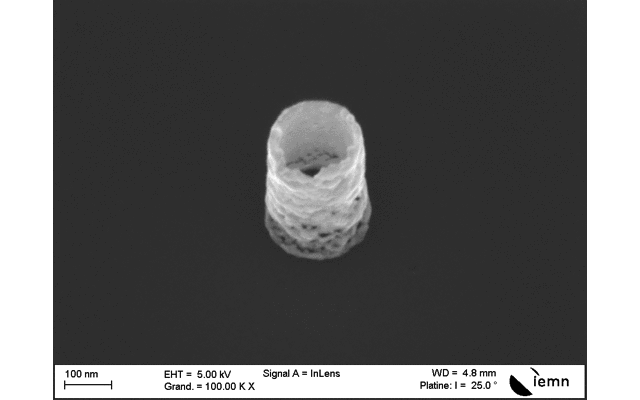

Supplement: Supplementary file 6 — 500nm 18deg [file 41598_2019_44074_MOESM6_ESM.gif]

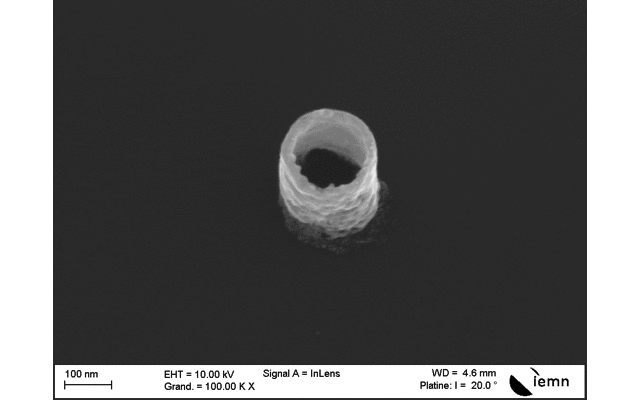

Supplement: Supplementary file 7 — 500nm 21deg [file 41598_2019_44074_MOESM7_ESM.gif]

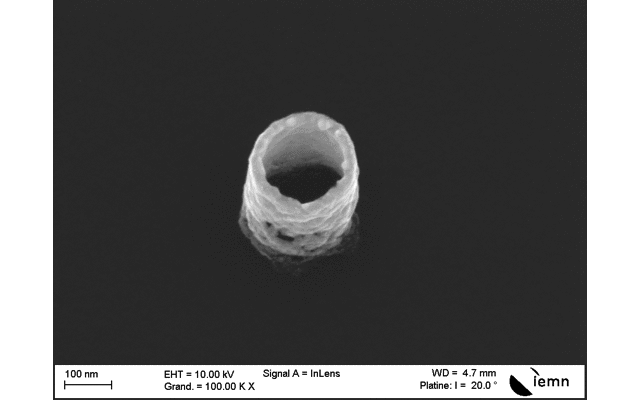

Supplement: Supplementary file 8 — 500nm 24deg [file 41598_2019_44074_MOESM8_ESM.gif]

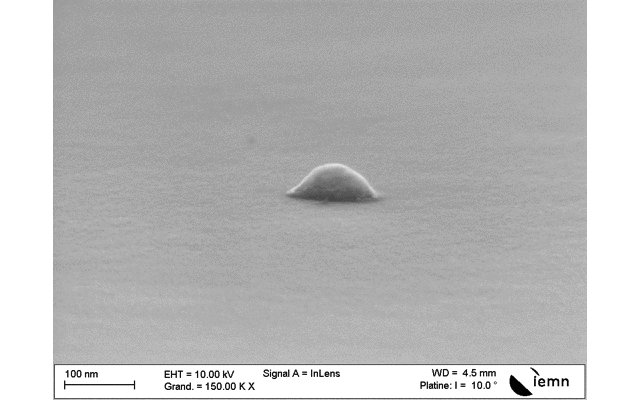

Supplement: Supplementary file 9 — 950nm 1deg [file 41598_2019_44074_MOESM9_ESM.gif]

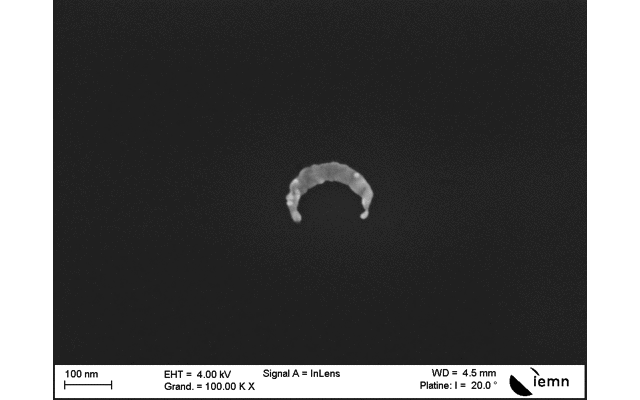

Supplement: Supplementary file 10 — 950nm 6deg [file 41598_2019_44074_MOESM10_ESM.gif]

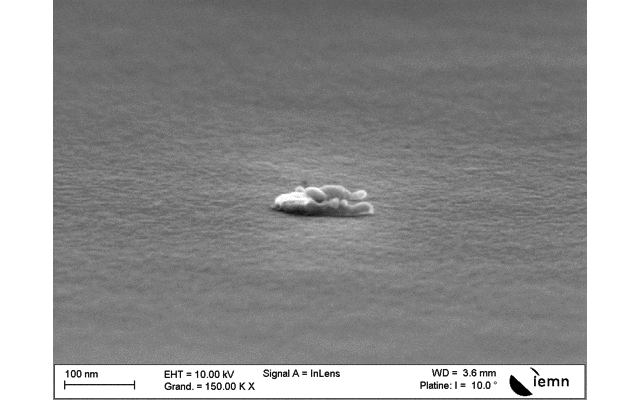

Supplement: Supplementary file 11 — 950nm 6deg_1 [file 41598_2019_44074_MOESM11_ESM.gif]

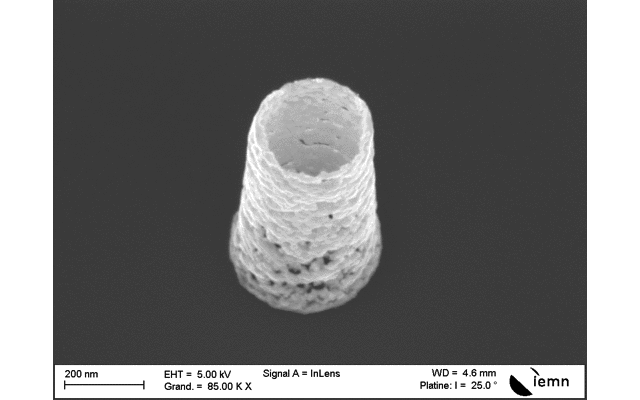

Supplement: Supplementary file 12 — 950nm 15deg [file 41598_2019_44074_MOESM12_ESM.gif]

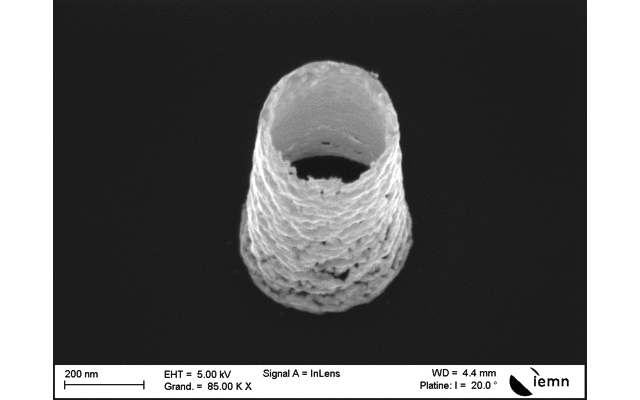

Supplement: Supplementary file 13 — 950nm 18deg [file 41598_2019_44074_MOESM13_ESM.gif]

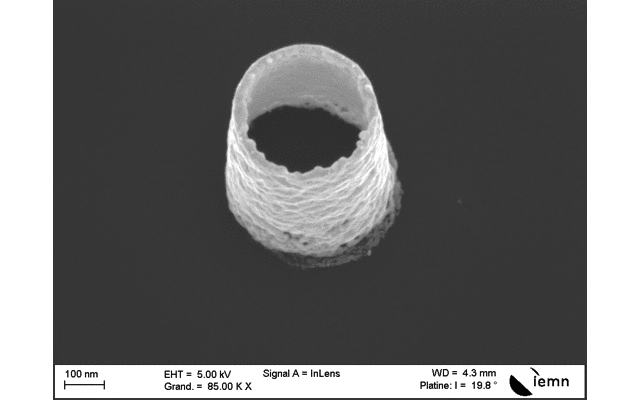

Supplement: Supplementary file 14 — 950nm 21deg [file 41598_2019_44074_MOESM14_ESM.gif]

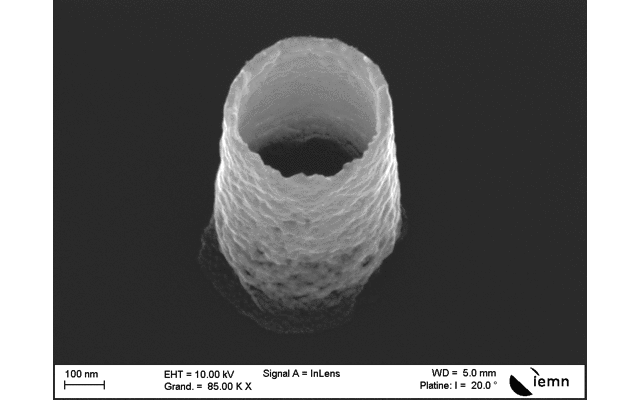

Supplement: Supplementary file 15 — 950nm 24deg [file 41598_2019_44074_MOESM15_ESM.gif]
